# Mereological Anti-Conservatism

**DOI:** 10.1007/s12136-025-00630-w

**Published:** 2025-02-26

**Authors:** Alexandre Declos, Vincent Grandjean

**Affiliations:** 1 University of Neuchâtel, Neuchâtel, Switzerland; 2 University of Zurich, Zurich, Switzerland

**Keywords:** Mereology, Special composition question, Conservatism, Ordinary objects, Arbitrary parts

## Abstract

In this paper, we examine an overlooked answer to the Special Composition Question (SCQ), termed “Mereological Anti-Conservatism.” This view posits that extraordinary objects exist but that ordinary objects do not. For example, while tables and chairs do not exist, the mereological sums of these items do correspond to real objects. Although such a claim may initially seem absurd, we argue that (i) it is entirely derived from the claims and commitments of traditional rival theories—Nihilism, Universalism, and Conservatism; (ii) it resolves several issues that plague Conservatism, such as problems of vagueness, change and persistence, and the shortcomings of common sense; and (iii) it offers a more plausible perspective than Conservatism when considering the vast scale of possible worlds with differently segmented realities. Ultimately, we contend that if Anti-Conservatism is deemed implausible, then Conservatism must be as well, for structurally similar reasons.

## Introduction

Do ordinary objects like chairs exist or are there merely particles arranged chair-wise? And what about extraordinary objects, such as one composed of a chair and the Taj Mahal—does *that* exist? These questions, which have been at the heart of intense metaphysical debate over the last decades, fall under the “Special Composition Question” (SCQ). The SCQ asks whether composite objects—i.e., objects with proper parts—exist. Traditionally, three answers have been proposed to the SCQ: Nihilism, Universalism, and Conservatism. The first two are considered “revisionary,” while the latter is deemed “common-sensical.”

In this paper, we introduce and explore a fourth answer to the SCQ: Anti-Conservatism. This view, constructed entirely from claims and commitments of the traditional answers, has yet to be identified within the logical space. The outline is as follows. First, we present Anti-Conservatism as a neglected alternative to the traditional answers to the SCQ and provide a clear formulation of the view (§2). Second, we demonstrate that Anti-Conservatism can resolve several issues encountered by Conservative ontologies, including problems raised by vagueness, change and persistence, and the shortcomings of common sense (§3). Third, we show that Anti-Conservatism can be supported by a modal argument, highlighting its plausibility when considering the vast scale of possible worlds with differently segmented realities (§4). Fourth, we address several objections against the viability of Anti-Conservatism, including the mysterious non-existence of objects composed of existing parts, the demiurgic power of the human mind, and the specific status of organisms (§5). Finally, we argue that the strongest objections to Anti-Conservatism apply, *mutatis mutandis*, to Conservatism. Thus, Anti-Conservatism serves as a lens that reveals the fundamental problems inherent in Conservative ontologies (§6).

Before proceeding, let us make an important disclaimer: we do *not* endorse Anti-Conservatism in this paper. Rather, our aim is to demonstrate that if one finds Anti-Conservatism untenable (as we do, and as we anticipate most readers will), then one has even stronger grounds to reject Conservatism. In other words, Anti-Conservatism is introduced here as a purely dialectical tool, serving to expose the profound flaws inherent in Conservatism.

## Anti-Conservatism Within the Logical Space

The metaphysics of material objects begins with determining which material objects exist. This inquiry can be approached via mereology, especially through the “Special Composition Question”: when do some objects compose something else? (cf. Van Inwagen, 1990: §12). Traditionally, three answers have been proposed to the SCQ: two “revisionary” answers and one “common-sensical” answer.^1^

The first revisionary answer is *never*. No objects would ever compose anything. This view, known as “Mereological Nihilism,” asserts that there are no composite objects such as chairs, cars, or the composite of a chair and the Taj Mahal. Instead, there are only simples—entities, whether extended or not, that lack proper parts—arranged chair-wise, car-wise, or chair-and-the-Taj-Mahal-wise. Nihilism, however, is seldom defended in its strictest form. Most proponents allow exceptions for persons and other living organisms, a concession that gives rise to the less radical stance known as “Organicism” (cf. Merricks, 2001; Van Inwagen, 1990).

Opposite to Nihilism, yet equally revisionary, is “Mereological Universalism.” Proponents of this view answer the SCQ with *always*. Any collection of objects whatsoever would compose a further object. On this view, not only do tables, cars, mountains, trout, and turkeys exist, but so do a plethora of “extraordinary” composite objects, such as the mereological sums of tables and cars, mountains and tables, and even trout and turkeys (Lewis, 1991). This ontological framework is supported by Classical Extensional Mereology (CEM), which endorses “unrestricted composition” as an axiom (cf. Cotnoir & Varzi, 2021: 30).

Between these two revisionist views lies an intermediate answer: *sometimes*. Some pluralities of objects, but not all, would compose a further object. This type of answer to the SQC maintains that composition occurs (contra Nihilism) but that it is restricted (contra Universalism). Such “Restrictivism” relies on the general schema of the form: “the *xs* compose *y* iff condition φ is satisfied.” The condition φ can, of course, be specified in various ways. For instance, one might argue that composition occurs when a plurality of objects shares a specific kind of physical bond.^2^ Alternatively, one could appeal to physical principles, claiming that composition happens when objects are in a bound state (Calosi, 2022). In fact, it is easy to imagine a vast array of arbitrary or eccentric intermediate answers to the SCQ—such as the view that the *xs* compose only when their number is prime. However, among all these possibilities and variants of Restrictivism, one view has become particularly prominent in the literature: “Mereological Conservatism.” This view seeks to align with common sense beliefs about existence. It asserts that objects like chairs, tables, and the Taj Mahal exist while dismissing entities like trout-turkeys or the composite of the Eiffel Tower and Mike Tyson’s nose as metaphysical fantasies. Although providing a precise definition of “ordinary objects” is notoriously challenging, they can be approximated as “[…] objects belonging to kinds that we are naturally inclined to regard as having instances based on our perceptual experiences” (Korman, 2020: §1.1).

This usual framing of the SCQ debate in terms of extreme and moderate answers has some limitations, however. One is its failure to clearly distinguish the specific commitments each view holds regarding the existence of ordinary versus extraordinary objects. This ambiguity leads to two significant issues: (i) it does not explain why “Mereological Conservatism” appears prima facie attractive and (ii) it conceals the variety of possible views within the *sometimes* position, including what we shall call Anti-Conservatism. To address these issues, we propose a reformulation of the debate, mapping answers to the SCQ based on their commitments regarding ordinary and extraordinary objects^3^:

- **(i) Nihilism:** Composite objects, whether ordinary or extraordinary, do not exist.
- **(ii) Universalism:** Any composite object, whether ordinary or extraordinary, does exist.
- **(iii) Conservatism:** Only ordinary objects exist; extraordinary objects do not.

This new formulation reveals that the logical space is not exhausted by these three options. Another possibility, which to our knowledge has never been mentioned nor defended, is “Anti-Conservatism”:

- **(iv) Anti-Conservatism:** Only extraordinary objects exist; ordinary objects do not.

For instance, Anti-Conservatism posits that there are no tables, mountains, or cars but accepts the existence of simples arranged table-wise and of mereological composites of tables and mountains. At first, such a claim might seem absurd. Why would anyone endorse a view that denies familiar objects only to introduce a myriad of strange entities we have no awareness of—and no use for—in our daily lives? Consequently, one might object that Anti-Conservatism is entirely ad hoc. While this view is a possible answer to the SCQ, it seems gratuitous and groundless.

However, this objection does not carry much weight. Anti-Conservatism is not without ground, insofar as it functions like a *dialectic vampire*, that merely recombines claims and commitments that have been independently defended by Nihilists, Universalists, and Conservatists.

To begin with, Anti-Conservatists reject the existence of ordinary objects, much like Nihilists. At first glance, they can draw upon well-known Nihilist arguments, such as the overdetermination argument, which posits that admitting composites in addition to their atomic parts leads to causal redundancy (Merricks, 2001). They can also utilize the argument from explanatory benefits, which suggests that dispensing with ordinary objects resolves many vexing philosophical puzzles.

Moreover, Anti-Conservatists can draw on arguments put forth by Universalists to support the existence of extraordinary objects. For instance, the argument from arbitrariness (Korman, 2011: §8) asserts that there is no significant ontological difference between certain instances of ordinary objects (e.g., islands, galaxies) and extraordinary ones (e.g., chables, fusions of trouts and turkeys). Anti-Conservatists can also invoke the debunking argument (Korman, 2011: §7), which contends that our beliefs about what exists are largely the product of cultural or biological contingencies.

**Fig. 1:**
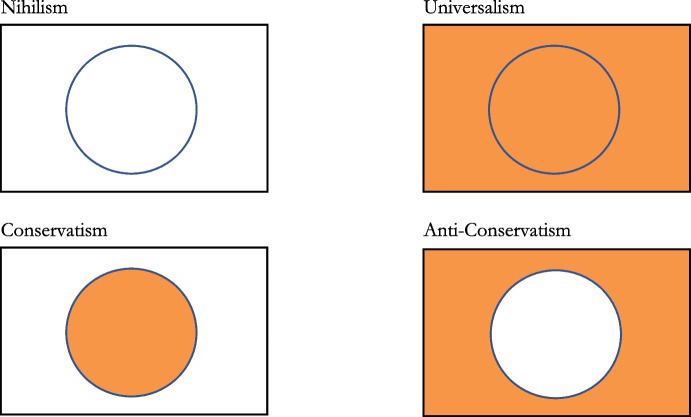
The logical space. The rectangle represents the universe of all composite objects. The circle denotes the set of ordinary objects within that universe. The zone outside the circle corresponds to the set of extraordinary objects within that universe. The orange color indicates which areas contain existing objects

Lastly, Anti-Conservatists agree with Conservatists that a distinction must be drawn between ordinary and extraordinary objects. While we ordinarily believe in the existence of chairs and tables but not chables, Anti-Conservatists acknowledge this intuitive distinction, albeit to point out that we are fundamentally mistaken in our beliefs. They also concur with Conservatists that the correct answer to the SCQ is neither *never* (Nihilism) nor *always* (Universalism) but *sometimes*: not all pluralities of entities compose a further object. The situation is illustrated in Fig. 1.

The upshot is that Anti-Conservatism functions as a *dialectic vampire*: it is constructed entirely from claims already present in rival accounts and can freely draw upon the standard arguments advanced in favor of those claims. Of course, this does not provide a positive reason to endorse Anti-Conservatism. The fact that claims *x*, *y*, and *z* have been independently supported through various arguments does not, in itself, support the conjunctive claim *x* + *y* + *z*. However, the fact that Anti-Conservatism merely recombines arguments that have already been defended elsewhere shows that it is not entirely ad hoc.

## Could Anti-Conservatism Outperform Its Rivals?

The mere fact that Anti-Conservatism offers a possible answer to the SCQ does not inherently make it a compelling or viable solution. After all, who would seriously claim that chairs and tables do not exist yet advocate for the existence of “chables” (i.e., mereological fusions of chairs and tables)? Surprisingly, however, there are several compelling reasons to defend this position. Let us now explore them.

### Vagueness

One of the main challenges faced by Conservatists is the “argument from vagueness” (Lewis, 1986; Sider, 2001; see the discussion in Korman, 2020: §2.2). The argument is roughly as follows: if composition only occurs *sometimes*, then either (i) there are two almost identical material scenarios where composition occurs in one but not the other or (ii) there are borderline cases of composition, where it is indeterminate whether composition occurs. Option (i) faces a charge of arbitrariness: why would a collection of particles α compose something (e.g., a table) when arranged in a particular fashion but fail to do so if a single particle within α were slightly relocated? Option (ii) is problematic because it commits Conservatists to *ontic vagueness*, implying that it is indeterminate how many objects exist. Consequently, it seems preferable to think that composition either *never* or *always* occurs, thus supporting either Nihilism or Universalism.

Our interest here is not in how Conservatism might respond to this challenge (see Korman, 2011: ch. 9), but rather that it faces this challenge while rival views do not. Nihilism states that there are no composites, thus sidestepping the issue of when composition occurs. Universalism removes the threat of arbitrariness or vagueness by positing that any arrangement of objects composes something. Anti-Conservatism seems to share a similar advantage, as it only acknowledges extraordinary objects, which are extensionally defined. The argument from vagueness, after all, targets *ordinary* objects, not extraordinary ones. By dismissing ordinary objects entirely, Anti-Conservatism therefore seems able to avoid the argument from vagueness, much like Universalism and Nihilism.

Unfortunately, things are not so simple. As the mirror image of Conservatism, Anti-Conservatism also requires a demarcation between extraordinary and ordinary objects or between cases of composition and non-composition. This demarcation reintroduces the vagueness problem. Consider the fusion *F* of a chair and an anvil. For Anti-Conservatism, *F* exists. Removing one atom from *F* results in a distinct extraordinary object *F′*. Repeating this process leads to *F″* and so on, until we reach a collection of atoms corresponding to what we would ordinarily consider a chair. For Anti-Conservatism, *that* collection composes nothing. Thus, a line must exist somewhere between composition and non-composition, raising questions of arbitrariness or indeterminacy. If Conservatism struggles to explain when a chair starts and stops being composed, Anti-Conservatism faces a parallel difficulty.

However, while Anti-Conservatism is not entirely free from vagueness concerns, these issues are far less pervasive than in Conservatism. The argument from vagueness poses only a minimal threat to Anti-Conservatism, because it only applies to collections of particles that we mistakenly consider real—viz., to ordinary objects. These collections represent an insignificant fraction compared to the vast array of extraordinary objects in the Anti-Conservatist ontology. In contrast, the argument from vagueness permeates the entirety of Conservatism’s ontology, where ordinary objects are the only objects that exist. In other words, while vagueness problems affect only a small portion of Anti-Conservatism’s ontology, they account for 100% of Conservatism’s ontology. Thus, Anti-Conservatism largely sidesteps the argument from vagueness, as composition *almost always* occurs under this view. Although this does not provide a reason to prefer Anti-Conservatism over Universalism or Nihilism (both of which avoid the vagueness problem entirely and offer more straightforward explanations), it does offer a compelling reason to favor Anti-Conservatism over Conservatism.

Some might counter that, in philosophy, the frequency of a problem is irrelevant—vagueness remains a concern regardless of how often it arises. To this, we offer an analogy: imagine two furniture factories, each producing 1000 defective tables per year. If one factory’s entire output is defective, while the other produces 10,000 tables annually, which factory would you trust for your next purchase? The same reasoning applies to Conservatism and Anti-Conservatism: we do not claim that Anti-Conservatism entirely avoids the vagueness problem or that it has fewer vague instances than Conservatism. Rather, we argue that the problem arises far less frequently in its ontology, providing a strong reason to prefer Anti-Conservatism to Conservatism.^4^

### Change and Persistence

Ordinary objects give rise to many infamous puzzles: is Theseus’ ship the same ship once all its original parts have been replaced? Is a dismantled watch on a workshop table still a watch? How many grains of sand can a heap of sand lose before ceasing to be a heap? If a clay statue cannot survive being flattened into a disk, while the lump of clay that constitutes it can, does not this imply that two distinct objects coincide where we ordinarily perceive only one? What kinds of change, if any, can ordinary objects (such as statues, mountains, clouds, and cars) survive?

Conservatism must grapple with all these puzzles, while rival accounts do not. If, as Nihilists believe, there are only mereological simples, there is no need to worry about the Ship of Theseus, heaps of sand, or broken watches because there are no such things as ships, heaps, or watches (however, see Rettler, 2018). Universalists also handle these puzzles effectively. If any collection of entities whatsoever composes something, the parts that initially made up Theseus’ ship still compose an object (though perhaps not a ship) at later times, regardless of their location. Moreover, Universalists argue that whenever an old part is replaced by a new one, the previous object is replaced by another, numerically different one. Determining which of these entities deserves to be called “the Ship of Theseus” becomes a conceptual or linguistic issue, not a metaphysical one (cf. Sider 200: 8–9). Universalists can address other issues surrounding the persistence conditions of ordinary objects in a similar manner.

Anti-Conservatism also sidesteps these puzzles concerning persistence and change.^5^ The reason is straightforward: these puzzles arise because of ordinary objects, and Anti-Conservatism simply denies the existence of such objects. Furthermore, these puzzles have no counterparts for extraordinary objects, which are defined purely extensionally. Removing or adding any atom from the fusion of the Eiffel Tower and the Moon results in another, distinct, extraordinary object. This means that extraordinary objects have all their parts essentially. While such Mereological Essentialism seems highly counterintuitive for ordinary objects, it is perfectly acceptable for arbitrary collections of entities or hunks of matter (van Cleve, 1986). Thus, one significant benefit of Anti-Conservatism is that it completely dispenses with familiar issues surrounding change and persistence. While this is not a reason to favor Anti-Conservatism over Nihilism and Universalism, it does provide another compelling reason to prefer it over Conservatism.

### The Shortcomings of Common Sense

Anti-Conservatism is an extremely revisionary view—arguably more so than either Universalism, which in some versions still acknowledges the existence of ordinary objects, or Nihilism, which denies ordinary objects but aligns with common sense by also rejecting *extraordinary* objects. In fact, no view could be more revisionary than Anti-Conservatism, as it claims the existence of everything we typically consider nonexistent, while denying the existence of everything we ordinarily recognize as existent.^6^

However, we contend that such a departure from common sense could be seen as a strength rather than a flaw. There are compelling reasons to question whether the world is truly as we perceive it and to doubt whether common sense serves as a reliable guide in metaphysical inquiry. Some argue that common sense verdicts are messy and uncertain, historically changing, or culturally variable (Rorty, 1989). Others note that common sense is riddled with biases and, at times, internally contradictory (Schwitzgebel, 2024). Even setting aside these issues, it is unclear why common sense beliefs should hold any privileged epistemic status in metaphysics. As Sider provocatively asks, “Why should the inherited prejudices of our forebears count for anything? It’s hard to imagine a greater abdication of the founding spirit of philosophy than the exhortation: ‘believe this because lots of other people do’” (2013: 246; see also Frances, 2021; Ladyman & Ross, 2007).

In fact, we have grounds to consider common sense highly unreliable. A quick look at the history of science shows that common sense beliefs have repeatedly failed us: “traditional commonsense folk opinion has proven radically wrong about central issues in physics, biology, and scientific cosmology” (Schwitzgebel, 2024: 19; see also Rinard, 2013; Ladyman & Ross, 2007: 11). This is particularly evident in the philosophy of time: common sense suggests the existence of a unique present, yet “[Relativity] states that there is no absolute relation of objective simultaneity: two spatially separated events may be simultaneous for one observer, and temporally distant for another” (Grandjean, 2022: 2). These suspicions extend to our ordinary beliefs about material objects. We might think there is a table and a chair but no “chable” in the room. However, how confident can we be in this belief? While we perceive the table as a solid, spatially continuous object, physics reveals it to be a swarm of moving particles in mostly empty space. The sharp boundaries we see at the macroscopic level appear, upon closer inspection, to have “the same degree of arbitrariness as that of any mathematical graph smoothed out of scattered and inexact data, the same degree of idealization of a drawing obtained by ‘connecting the dots’, the same degree of abstraction as the figures’ contours in a Seurat painting” (Varzi, 2014: 28; see also Fairchild & Hawthorne, 2018). While none of this conclusively proves that there are no tables or that there are chables, it casts doubt on the reliability and relevance of common sense for metaphysical inquiry. As Sider notes, “[…] there is no independent reason to think that common sense is reliable about whether there exist tables and chairs as opposed to there merely existing suitably arranged particles. Our forebears presumably did not even consider the latter possibility” (2013: 246).

Conservatists might argue that ordinary objects of common sense are very different from the extraordinary objects of Anti-Conservatism (and Universalism). They claim the former exhibit “[…] a kind of unity and causal covariance that is altogether lacking in the latter” (Korman, 2011: 139). However, many alleged ordinary objects—galaxies, bikinis, decks of cards, archipelagos, and assortments—seem just as scattered, qualitatively heterogeneous, and causally disjointed as chables. Additionally, relying on perceptual evidence to support the existence of tables and chairs offers little support. Opponents to Conservatism can argue that our perceptual experiences would be identical if there were particles arranged chair-wise composing nothing (Merricks, 2001), if there were chables alongside tables and chairs, or if there were only chables and no tables or chairs. Even our best science would remain unaffected by the truth of any of these views.

Anti-Conservatism is certainly a strange view. But why would this be a problem? According to Schwitzgebel (2024), all extant answers to fundamental metaphysical problems, such as the relation between mind and matter, are both bizarre (contrary to common sense) and dubious (they do not compel belief). A similar point may apply to answers to the SCQ, which all involve counterintuitive claims or consequences. Nihilists and Anti-Conservatists claim there are no chairs. Universalists and Anti-Conservatists claim there are chables. Conservatists and Anti-Conservatists assert that the world is such that the tiniest material difference can bring new entities into being. They also hold the bizarre view that our common picture of the world is, quite miraculously, either exactly fitted to what there is (Conservatism) or to what there is not (Anti-Conservatism). Thus, we can adopt Schwitzgebel’s argument: while we do not know which of these theories is true, we know that one of them (or a variant) must be true, as they exhaust the spectrum of possible answers to the SCQ. Yet each of these options violates common sense to some extent. Therefore, no metaphysics of composition can fully align with common sense. Highlighting that a view conflicts with common sense carries little weight, as they all do.

The revisionary nature of Anti-Conservatism, as we argued, does not qualify as a reason to reject that view. While Conservatism defends our common sense picture of the world, Anti-Conservatism asserts that the world is nothing like we represent it and can therefore take the critiques against common sense at face value. In fact, objections to common sense that undermine the plausibility of Conservatism may simultaneously bolster the plausibility of rival views, including Anti-Conservatism. If we have good reasons to believe that the world is not as we represent it, any view that diverges from that common picture may have a better chance of being correct.

Before proceeding, two potential objections must be addressed. The first is that Anti-Conservatism cannot consistently dismiss the value of common sense, as it relies on a common sense distinction between extraordinary and ordinary objects (after all, the concept of an “ordinary object” seems to be rooted in common sense thinking). The second objection is that it is illegitimate to invoke the history of science to argue, as we have above, that common sense judgments are generally unreliable in metaphysical matters. Some might indeed argue that this parallel is flawed. For instance, Amie L. Thomasson notes, “[…] the reason scientific theories sometimes warrant dismissing common-sense views is that scientific theories may be empirically extremely well confirmed, giving us strong reason to believe their direct consequences, even if they conflict with prior widely held or ‘common-sense’ beliefs. But the same epistemic status may not be shared by philosophical theories” (2004: 84). In other words, while scientific evidence or well-confirmed theories may undermine common sense beliefs, this does not provide a principled reason to exclude common sense from the metaphysical inquiry. In fact, it is unclear whether metaphysical theories can be empirically confirmed at all or what sort of evidence would even apply. Therefore, common sense arguably remains central (and perhaps the only available standard) in “pure” metaphysical debates, such as those concerning the SCQ.

In response to the first objection, it is worth noting that this concern applies to other revisionary views as well. For instance, although Nihilists deny the existence of ordinary objects, they still rely on ordinary object concepts (such as “table” and “chair”) to articulate their view. To argue that there are no chairs, only simples “arranged chairwise,” Nihilists must still reference the concept of a chair to make their position intelligible. The same applies to Anti-Conservatism, which uses the concept of “ordinary objects” even while rejecting their existence. Crucially, however, the distinction between ordinary and extraordinary objects is employed here solely as a heuristic device, valuable for reshaping the available answers to the SCQ and positioning Anti-Conservatism as a legitimate alternative. This distinction serves to clarify the theoretical options but does not suggest that common sense has any special epistemic authority.

In response to the second objection, we argue that it only holds if one assumes a strict separation between science and metaphysics—namely, that there are “pure” metaphysical debates entirely independent of scientific inquiry. The problem is that this assumption does not apply to all metaphysical discussions. For example, in the philosophy of time, one cannot simply ignore physical theories and evidence in favor of relying solely on common sense beliefs about time. If a temporal ontology conflicts with relativity, this presents a serious issue that must be addressed (Grandjean, 2022: 5). Similarly, we argue that the debates surrounding material composition are not entirely detached from scientific inquiry. In fact, it is possible to argue that our best physical theories should inform attempts to answer the SCQ (Calosi, 2022). If that is the case, then the unreliability of common sense in scientific matters may also undermine its authority in metaphysical debates, particularly those involving material composition.

## The Modal Argument

So far, we have demonstrated that Anti-Conservatism is better equipped than Conservatism to address the traditional challenges posed by the metaphysics of material objects. However, is there a novel argument that could establish the superiority of Anti-Conservatism over Conservative ontologies? We believe there is one such argument—the modal argument—which we will present now.

It seems quite plausible that we could have carved out the world differently. We demarcate certain regions of space as islands because they are (at least sometimes) above sea level and could support human habitation. But could not we have stipulated instead that any portion of land less than 85% of the time above sea level is no island? Or that all land above sea level is, no matter what, an island? Or that islands come in and go out of existence with the tides? All these options, and many more, would have resulted in a different objectual carving. If Goodman (1955) is right, the point extends to virtually everything: we could have carved up the world in terms of grue and bleen things, emeroses and emerubies, and used countless other classifications undreamt of in any philosophy. At any rate, it does not seem inconceivable nor metaphysically impossible that other types of creatures would spontaneously see chables where we see chairs and tables (such creatures would perhaps need to have a very different cognitive and perceptual apparatus, but that is another matter).

Entertaining the thought of such alternative carvings is not just pure metaphysical speculation. Various philosophers have argued that scientific taxonomies and classifications could have been other than what they currently are. Chemists have judged that heavy water (D_2_O) is a variety of water (H_2_O), but some think that we would have been equally legitimate to conclude otherwise (LaPorte, 2004). Others insist that we could also have drawn the line between biological species differently than we do (Dupré, 1983).

Regardless of whether carving the world differently would have been correct, it seems quite clear that we *could* have carved out the world differently. We could have recognized more or fewer objects in reality or at least different objects than those we currently accept. This possibility gives rise to what we might call a “modal argument” for Anti-Conservatism. This argument highlights that Anti-Conservatism is more plausible than Conservatism across the scale of all possible worlds.

Suppose we had carved out the world differently, in terms of chables rather than chairs and tables. If Nihilism is true, doing so would have been a mistake, but no more so than believing there are tables and chairs. If Universalism is true, doing so would not have been a mistake, as there are indeed chables (and perhaps also tables and chairs). If Conservatism is true, carving the world differently would have been a mistake—for there are chairs and tables, but not chables. If Anti-Conservatism is true, this alternative carving would not have been a mistake—for there are chables but no chairs and no tables. Assuming that metaphysical views about composition aim to be necessarily true, these verdicts extend to all possible worlds.

Generalizing, Conservatism implies that the only true carving is the one we have adopted in the actual world and that is found in worlds where we (or our counterparts) or other creatures carve exactly as we do. Given that worlds with alternative carvings vastly outnumber those with a carving identical to ours, very few individuals get it right on the scale of all possible worlds. Epistemically speaking, we are in the best possible world (or one of the few best possible worlds): we always get it right when identifying material objects, while any world where people carve differently get it wrong.

With Anti-Conservatism, the situation is exactly reversed. The only incorrect carving is the one we have adopted in the actual world and that is found in any world where we (or our counterparts) or other creatures carve reality exactly as we currently do. Given that worlds with alternative carvings vastly outnumber those with a carving identical to ours,^7^ very few individuals get it wrong on the scale of all possible worlds. Epistemically speaking, we are in the worst possible world (or one of the few worst possible worlds): we always get it wrong when identifying material objects, while any world where people carve differently gets it right.

The consequence is that Anti-Conservatism is epistemically virtuous across possible worlds. On this view, while we are unlucky enough to be always wrong about what objects exist, virtually all denizens of other worlds get it right. Conservatism has the reverse consequence: while we are lucky enough to be always correct about which objects exist, virtually all denizens of other worlds are mistaken. In other words, Conservatism maximizes false beliefs about material objects across possible worlds, whereas Anti-Conservatism maximizes true beliefs about them. If we couple this thought with the seemingly reasonable premise that at least some other objectual carvings would have been equally acceptable, we have a reason to prefer Anti-Conservatism over Conservatism. Conservatism is a highly chauvinistic view, implying that anything diverging from our actual beliefs is a mistake. Those appalled by this idea should find Anti-Conservatism more appealing, as it allows for many alternative carvings—in fact, nearly all of them—to be equally valid. (Again, this does not demonstrate that Anti-Conservatism is true or that it is superior to Universalism or Nihilism, but it does suggest that it is preferable to Conservatism.)

Before proceeding, we must address two potential objections to the foregoing modal argument.

First, some could argue that what counts as “extraordinary” versus “ordinary” depends on the world in question. In the actual world, chairs and tables are considered ordinary objects, whereas chables are not. But could not there be worlds where the reverse holds—worlds in which people naturally believe that chables exist, but that chairs and tables do not? If this were the case, the modal argument would collapse. The reason is straightforward: the classification of carvings as “correct” or “incorrect” would then depend on the conceptual schemes of each world, leaving Anti-Conservatism with no principled advantage over Conservatism across possible worlds.

Our response is that this objection rests on a misunderstanding. Specifically, it relies on an *intensional* interpretation of Anti-Conservatism, where the classification of “ordinary” and “extraordinary” objects varies across worlds. We categorically reject this interpretation. Instead, the modal argument adopts an *extensional* reading, where the reference of “ordinary” and “extraordinary” objects is fixed across all possible worlds. Put otherwise, we maintain that the sets of extraordinary and ordinary objects are the same in every world. Chairs and tables are always ordinary objects, while chables are always extraordinary. The classifications are independent of any conceptual schemes that might arise within a particular world.

The intensional interpretation must also be rejected for a more fundamental reason: it leads to absurd consequences for both Conservatism and Anti-Conservatism. If this interpretation were accepted, Conservatists would be forced to admit that chables are “ordinary” in some worlds and thus exist in those worlds, even if they do not exist in ours. Similarly, Anti-Conservatists would have to concede that chairs are “extraordinary” in certain worlds and thus exist in those contexts, even though they reject chairs outright in the actual world. These conclusions undermine the coherence of both views, as neither Conservatists nor Anti-Conservatists would accept such fluid commitments about the existence of these objects.

A second potential objection concerns our claim that Conservatism is a “chauvinistic” view. Some could argue that the same criticism applies to Anti-Conservatism. After all, Anti-Conservatism also grants a metaphysical privilege to the actual world (and to worlds with identical carvings), just as Conservatism does. In both cases, beliefs about what is ordinary and extraordinary in the actual world (or in worlds with identical carvings) determine what exists across all possible worlds. Consequently, one might contend that if Anti-Conservatism is chauvinistic, so too is Conservatism, for essentially the same reason.

Our response to this concern is straightforward. We do not claim that Anti-Conservatism is not chauvinistic at all, but rather that it is *less so* than Conservatism. In fact, we can concede the point that Anti-Conservatism, like Conservatism, confers a suspicious metaphysical privilege on the actual world and worlds with identical carvings. However, there remains a critical distinction. Anti-Conservatism allows for a vast array of alternative carvings—virtually all of them—to be correct ontologically speaking. The only mistaken carving, on this view, is the one chosen by us (or by people in worlds with identical carvings). This means that getting it wrong about material objects under Anti-Conservatism is exceedingly difficult: one simply needs to carve differently from us to be correct. With Conservatism, the situation is reversed: all alternative carvings are incorrect ontologically speaking; the only correct one is the one we (or people in worlds with identical carvings) have chosen. This makes it remarkably easy to get it wrong about material objects: any deviation from the actual carving (excluding worlds with identical carvings) qualifies as a mistake. As this comparison illustrates, Conservatism is far more chauvinistic than Anti-Conservatism. While both views grant a metaphysical privilege to the actual world, Conservatism uniquely positions its chosen carving as the sole correct one, dismissing all others as erroneous.

## Anti-Anti-Conservatism

In this section, we review some of the immediate objections that might be raised against the viability of the Anti-Conservative position.

### Sums and Parts

A significant objection concerns the status of extraordinary objects that are mereological sums of ordinary objects, such as “chables.” How does the Anti-Conservatist justify the existence of a chable while denying the existence of the specific table and chair from which it is composed? It seems problematic to claim that a fusion is real while maintaining that its parts are not. Yet Anti-Conservatism implies that when P and Q are ordinary objects, the fusion P + Q exists—provided that it is not itself ordinary—while neither P nor Q exists.

The situation appears analogous to that of organicists, like Van Inwagen (1990) and Merricks (2001), who assert that Descartes exists but that Descartes’ left leg, which is part of him, does not. Van Inwagen famously addresses this difficulty by rejecting the “Doctrine of Arbitrary Undetached Parts (DAUP),” according to which “for any material object M, if R is the region of space occupied by M at time *t*, and if sub-R is any occupiable sub-region of R, then a material object occupies sub-R at *t*” (Van Inwagen 1990: 123). Van Inwagen rejects DAUP because it logically implies a form of mereological essentialism he finds implausible, namely, “Mereological Near-Essentialism (MNE).” MNE states that “[…] if a part is removed from an object, and no new part is added to the ‘remainder’, then that object must therewith cease to exist” (Van Inwagen 1990: 124). Van Inwagen refutes MNE by using the example of Descartes, who could survive the loss of his left leg, thereby demonstrating that MNE and, consequently, DAUP are false. Furthermore, Van Inwagen argues that the presence in our terminology of an expression such as “Descartes’ left leg,” but not of “all of Descartes except his left leg,” is linguistically accidental and depends on our contingent interests.

Can a similar response be used to account for the ordinary parts of extraordinary objects in the Anti-Conservative ontology? At first glance, it seems unlikely, for two reasons. First, the ordinary parts of many extraordinary objects, such as chables, are detached. Second, while MNE seems implausible for ordinary objects, it appears much more plausible for extraordinary objects. For instance, Anti-Conservatists will maintain that if one of the eight legs that composes a chable were destroyed, the resulting (extraordinary) object would be numerically different (cf. §3, *Change and persistence*).

However, inspiration can be drawn from Van Inwagen’s solution to propose an original approach to the problem of *Sums and parts* in Anti-Conservatism. The Anti-Conservatist must reject DAUP when considering a special class of objects, namely, extraordinary objects with attached *ordinary* parts (e.g., the fusion of a vase and the chimney on which it sits). Furthermore, she must also reject the “Doctrine of Arbitrary *Detached* Parts (DADP),” which implies the existence of the detached table and chair that compose the chable. The rejection of both DAUP and DADP is based on the modal argument (§4), which suggests that identifying vases and chimneys, or tables and chairs, is relevant only in worlds carved like ours. On a vast scale of possible worlds, those ordinary objects have no functional role; therefore, affirming their existence merely because they are useful in our world is modally arbitrary and unjustified. In this sense, much like Van Inwagen argues for Descartes’ left leg, the categories of “vases,” “chimneys,” “tables,” and “chairs” appear accidental and depend on our contingent interests.

Does the rejection of DADP pose a logical problem? No, because for every extraordinary object—scattered or not—composed of ordinary parts, there are alternative carvings that allow the object to be redescribed as being composed of extraordinary parts. For example, a chable can be considered to have the following two parts: (i) a chair-and-tabletop and (ii) four table legs. Therefore, Anti-Conservatism remains logically coherent: existing extraordinary objects do have (extraordinary) existing parts.

### The Objection from Ordinariness

A second objection concerns the shifting nature of what is considered “ordinary” or “extraordinary” and its ontological implications. Take, for example, the spindle—a common tool used for spinning wool and linen in the Middle Ages. At that time, it was unquestionably an ordinary object. However, as centuries passed, the spindle has lost its ordinariness; today, only historians and a few traditional craftsmen can identify it. Does this mean, according to Anti-Conservatism, that the spindle has transitioned from non-existence to existence? Similarly, suppose that in 2100, fusions of fans and microwaves become essential tools for orientation on Mars. Would Anti-Conservatism then hold that these fusions cease to exist once they become functional? Such conclusions seem wildly implausible, as they would suggest that by merely categorizing entities as ordinary or extraordinary, we could bring them into or out of existence. This would seem to grant us demiurgic powers. But, the objection goes, objects themselves do not change intrinsically when we label them as “ordinary” or “extraordinary”; it is only our perception of them that shifts.

Interestingly, Conservatists face a similar issue: does the spindle cease to exist when it is no longer considered ordinary? Will the fan-microwave fusion come into existence once our descendants start using such objects and regard them as commonplace? To avoid this problem, both Anti-Conservatists and Conservatists must accept a principle stating that an object is ordinary if, at any point in human history, it is recognized as such (and is extraordinary otherwise). Thus, the spindle and the fusion of fans and microwaves were, are, and will always remain ordinary objects. Consequently, for Anti-Conservatists, these objects never exist, while for Conservatists, they always do. Objects that are ordinary or extraordinary, that is, are tenselessly so. This principle prevents the concept of intermittent existence, ensuring that the ontological status of material objects remains consistent regardless of changing perceptions.

### Persons and Organisms

A final objection to Anti-Conservatism concerns the status of organisms and persons. If we assume that these entities are ordinary objects, as they indeed appear to be, then Anti-Conservatism would imply that they do not exist. But this conclusion seems untenable: surely there are cats, cows, birds, and human beings—don’t *we* exist? Such a conclusion would be unacceptable to anyone but the most radical Nihilists, likely consigning Anti-Conservatism to the philosophical limbo where it perhaps belongs.

Several responses can be considered here. One approach would be to modify Anti-Conservatism to recognize that organisms and persons hold a special status and therefore warrant distinct treatment. According to this view, only extraordinary objects and a select few ordinary objects (specifically, organisms and persons) would exist. This strategy mirrors the move made by Organicists to avoid the extremes of full-blown Nihilism (van Inwagen, 1990), and it could, in principle, be applied here as well. However, this restriction seems arbitrary and should be avoided by Anti-Conservatism if possible.

Another possibility is to concede that there are no organisms and no persons, but to argue that other entities can fulfill the same roles. Anti-Conservatists do admit the existence of proper parts of persons (e.g., John minus one atom) and proper parts of organisms (e.g., Tib but not Tibbles). They also accept fusions of persons or organisms with other entities: for instance, the fusion of John with a speck of dust on his skin is considered an object. These extraordinary objects might be sufficient, for all practical purposes, to account for our everyday discourse about organisms and persons. They could be seen as “proxy” persons or organisms. (Note, however, that this view would face a version of the well-known “too-many thinkers” problem, as it leads to an overabundance of “proxy” persons, making it indeterminate which one corresponds to a given individual—such as which one is truly “me.”).

A third option, particularly regarding the status of persons (whether human or non-human), is to accept that persons do not have any parts at all, either because they are immaterial or because they supervene on a physical basis without themselves being composite entities. On this view, persons would not be subject to the SCQ, allowing Anti-Conservatism to avoid the claim that we—and all the people we know—simply do not exist.

Finally, one might attempt to classify organisms and persons as extraordinary objects, thus affirming their existence. However, this claim is difficult to justify within the framework of Anti-Conservatism. Extraordinary objects, as defined, are typically far removed from the ordinary and intuitive status of persons and organisms, making this move feel strained and ad hoc.

## Conclusion

The upshot of this discussion is that Anti-Conservatism, while undoubtedly a bizarre metaphysical view, is neither ad ho*c* nor indefensible. In fact, some of the points mentioned above might even provide reasons to prefer it to Conservatism. However, we acknowledge that none of what has been discussed serves as a direct argument for the truth of Anti-Conservatism. Indeed, we do not believe there is any compelling reason to favor this view over Nihilism or Universalism, which are more systematic and less objectionable.^8^ As such, we share what is likely the reader’s impression at this stage: Anti-Conservatism is highly implausible.

But here is the crucial point: if Anti-Conservatism is implausible, so too is Conservatism, and for precisely the same reasons. For instance, if you think Anti-Conservatism ought to be rejected because it introduces an ad hoc and arbitrary demarcation between cases where composition occurs and where it does not, then you should also reject Conservatism for the same reason. Similarly, if you think Anti-Conservatism is implausible because it would be a cosmic coincidence that the world happens to be exactly opposite to how we represent it, you should equally reject Conservatism, as it would be no less cosmic coincidence if the world happened to be exactly as we perceive it.

Therefore, while Anti-Conservatism may not be a plausible view, it has at least one significant merit: it highlights the shortcomings of Conservatism. And it is perhaps in this latter sense that our paper adopts an Anti-Conservatist stance. 9

## Footnotes

1 However, this classification is controversial. As we shall see, many Nihilists and Universalists argue that their views are entirely compatible with common sense.

2 For instance, Van Inwagen (1990) distinguishes between four ‘sometimes’ positions, characterized by an increasing strength of bond required for objects to compose a further object: *contact*, *fastening*, *cohesion*, and *fusion*.

3 The distinction between ‘ordinary’ and ‘extraordinary’ objects is quite common in the literature and is prima facie extensionally clear. That said, we acknowledge that this distinction is not essential for either Nihilism or Universalism. Some Universalists are happy to deny the existence of ordinary objects, such as tables and chairs. For instance, Heller (1990) treats physical objects as Quinean four-dimensional hunks of matter. Despite this complication, we maintain that our reframing of the answers to the SQC remains heuristically useful.

4 In fact, we believe that even Nihilism and Universalism encounter issues related to vagueness at some level. Nihilists must still account for when (and why) certain arrangements of simples qualify as being arranged “table-wise,” thereby corresponding to what we recognize as a table. Similarly, Universalists are tasked with explaining which specific mereological sums, among the many possible combinations, are captured by our ordinary concepts. Both Nihilists and Universalists might sidestep these concerns by adopting an epistemic or semantic account of vagueness. However, this approach would not address the argument from vagueness as it pertains to both Conservatism and Anti-Conservatism.

5 One notable exception is that Anti-Conservatism does not immediately resolve the problem of material constitution. While the puzzle of the statue and the clay does not arise for the Anti-Conservatist—simply because there are no statues on that view—an analogous issue persists with respect to extraordinary objects. For example, the relationship between a chable and the wood it is made of virtually raises the same worry as in the initial puzzle: these entities seem to have different (modal, historical, essential) properties while being co-located. If pressed on this issue, one could adopt a classical constitution-based solution: if both the chable and the wood it is made of are considered extraordinary objects, they coexist and are co-located.

6 However, it is worth noting that the accusation of being “revisionary” against Anti-Conservatism only applies under a specific interpretation of what it means to be “revisionary.” If the revisionary nature of a position on the SCQ is measured by how many ordinary objects it accepts and how many extraordinary objects it rejects, then Anti-Conservatism is indeed maximally revisionary. But one might contend that this is not the only possible criterion. Another criterion could define a revisionary position as one that rejects the distinction between ordinary and extraordinary objects. By this measure, Anti-Conservatism would not be revisionary at all—especially in comparison to Nihilism and Universalism.

7 Some might object that there is an infinity of possible worlds where people carve reality exactly as we do, and therefore, worlds with alternative carvings cannot outnumber those with identical carvings. However, it is well established that one infinity can be greater than another, and there are far more ways to carve reality differently than identically. The infinity of worlds with different carvings thus surpasses the infinity of worlds with the same carving as ours. While this precision changes the arithmetic, it does not undermine the underlying argument.

8 A major reason Anti-Conservatism seems less appealing than Nihilism is its inability to effectively utilize the overdetermination argument—widely regarded as one of the strongest arguments in favor of Nihilism. According to Anti-Conservatism, both the chable and the particles arranged chable-wise are said to exist. For this reason, Anti-Conservatism also faces a threat of overdetermination. It should also be noted that rejecting composites of simples (e.g., particles arranged chable-wise) is not a viable option within its framework. Such a rejection would necessitate categorizing these composites as *ordinary* objects, which would paradoxically redefine Nihilism as a a form of Conservatism—one that accepts the existence of only ordinary objects, including particles arranged chable-wise. This result is clearly untenable and highlights a critical weakness in Anti-Conservatism’s ability to engage with one of the central arguments that make Nihilism compelling. 

9 An earlier version of this paper was presented at the Groupe d’Études en Métaphysique in Paris, and we are grateful for the valuable feedback received on that occasion. Additionally, we extend our sincere thanks to Guillaume Bucchioni, ClaudioCalosi, Francesco Franda, Uriah Kriegel, Anna Marmodoro, and Antoine Taillard for their insightful comments and suggestions. This research was supported by the SNSF Return Grant No. P5R5PH_222045, Time Regained, Part II: EpistemologicalPerspectives, based at the University of Zurich.
